# ﻿Two new species and two unrecorded species of Limacodidae (Lepidoptera, Zygaenoidea) from Xizang, China

**DOI:** 10.3897/zookeys.1100.76142

**Published:** 2022-05-12

**Authors:** Jun Wu, Alexey V. Solovyev, Hui-Lin Han

**Affiliations:** 1 School of Forestry, Northeast Forestry University, Harbin, 150040, China Northeast Forestry University Harbin China; 2 Department of Biology and Chemistry, Ulyanovsk State Pedagogical University, Ulyanovsk, 432071, Russia Ulyanovsk State Pedagogical University Ulyanovsk Russia; 3 Key Laboratory of Sustainable Forest Ecosystem Management, Ministry of Education, Northeast Forestry University, Harbin, 150040, China Northeast Forestry University Harbin China; 4 Northeast Asia Biodiversity Research Center, Northeast Forestry University, Harbin, 150040, China Ulyanovsk State Pedagogical University Ulyanovsk Russia

**Keywords:** Chongqing, new records, slug caterpillar moths, taxonomy, Tibet, Yunnan

## Abstract

Two new species (*Iragoidespeiwangi***sp. nov.** and *Caelestomorphaserratus***sp. nov.**) and two newly recorded species [*Euphlyctinaphaeopasta* (Hampson, 1906) and *Mummuaerata* Solovyev & Witt, 2009] are reported from China. Of these, the genera *Caelestomorpha* Solovyev & Witt, 2009 and *Mummu* Solovyev & Witt, 2009 are new to China; the female of the genus *Euphlyctina* Hering, 1931 is illustrated and described in this paper for the first time.

## ﻿Introduction

The slug caterpillar moths, namely the family Limacodidae of the superfamily Zygaenoidea, mainly occur in tropical and subtropical regions; the family Limacodidae contains 301 genera and 1672 species over the world (van Nieukerken et al. 2011). In recent years, with continued study of the Limacodidae fauna and discovery of a large number of new taxa (most of them from the Indo-Malayan and Palearctic realms), we are confident that the number of slug moths is nearly, or already more than, 1750 species to date (Solovyev et al. 2012; Solovyev and Saldaitis 2013, 2014; Wu and Chang 2013; Solovyev 2014, 2015, 2017; Ji et al. 2018; Lin et al. 2020; Wu et al. 2020, 2021a, 2021b, 2022; Nonaka 2021).

In this paper, we describe two new species and report two unrecorded species of the family Limacodidae from the Xizang Autonomous Region (= Tibet), China. These species belong to the following four genera: *Iragoides* Hering, 1931; *Caelestomorpha* Solovyev & Witt, 2009; *Euphlyctina* Hering, 1931; and *Mummu* Solovyev & Witt, 2009. Two genera, *Caelestomorpha* and *Mummu*, have not been previously reported in China. We review below each of the four genera included in this paper.

## ﻿Materials and methods

The specimens were collected with a 220V/450W mercury vapor light and a DC black light in the Xizang Autonomous Region, China. Wingspan was measured from forewing apex-apex and the forewing length from the wing base to the apex. Standard methods for dissection and preparation of the genitalia slides were used (Kononenko and Han 2007). The specimens were photographed using a Nikon D700 camera while the photographs of the genitalia slides were captured using an Olympus photo microscope aided by Helicon Focus software and then further processed using Adobe Photoshop CS6. The morphological terminology follows Epstein (1996).

All the type material of the new species and the examined newly recorded species are deposited in the collection of the Northeast Forestry University (**NEFU**), Harbin, China. Material from the Museum Witt München / Zoologische Staatssammlung München, Munich, Germany (**MWM/ZSM**) was also examined in this study.

### ﻿Abbreviations of institutes

**BMNH**The Natural History Museum, London, United Kingdom;

**MWM/ZSM** Museum Witt München/Zoologische Staatssammlung München, Munich, Germany;

**NEFU** Northeast Forestry University, Harbin, China;

**ZMHB** Zoologisches Museum der Humboldt Universität zu Berlin, Berlin, Germany.

## ﻿Taxonomic account

### ﻿Genus Iragoides

Taxon classificationAnimaliaLepidopteraLimacodidae

Hering, 1931

65602AB4-9D66-5E80-B7AE-F240074C2BDC

Iragoides Hering, 1931, in Seitz, Gross-Schmett. Erde 10: 671 (key), 709. Type species (original designation): Miresacrispa Swinhoe, 1890. Type locality India: Darjeeling.

#### Remarks.

The members of the genus *Iragoides* are of medium to large size, with a ground colour of reddish brown; the obvious silvery patches are always present on the foreleg and the base of the antennae. In the male genitalia, the uncus and gnathos are well developed; the juxta usually has a pair of finger-shaped lateral processes. *Iragoides* contains four known species to date: *I.crispa* (Swinhoe, 1890); *I.elongata* Hering, 1931; *I.dudai* Solovyev & Saldaitis, 2021; *I.nilgirica* (Hampson, 1891), three of which occur in China. *Iragoidesnilgirica* is known only from India (Wu and Fang 2008; Solovyev and Witt 2009; Solovyev and Saldaitis 2021).

In addition, Wu and Fang (2008), Solovyev and Witt (2009), and Solovyev and Saldaitis (2021) all presented the male adult of *Iragoideselongata* in their studies after it was described and illustrated with a hand-drawn adult by Hering (1931). Wu and Fang (2008) also listed both the male and female genitalia. However, as the original description of this species was relatively simple and lacked the description of genitalia, we will redescribe this species below.

### ﻿ Iragoides peiwangi sp. nov.

Taxon classificationAnimaliaLepidopteraLimacodidae

51CEF75B-0574-50FF-8C74-87DF1BAAF7D5

http://zoobank.org/B923CADB-4342-400B-9BBD-1C68CEA20099

**Figures 1–11.**
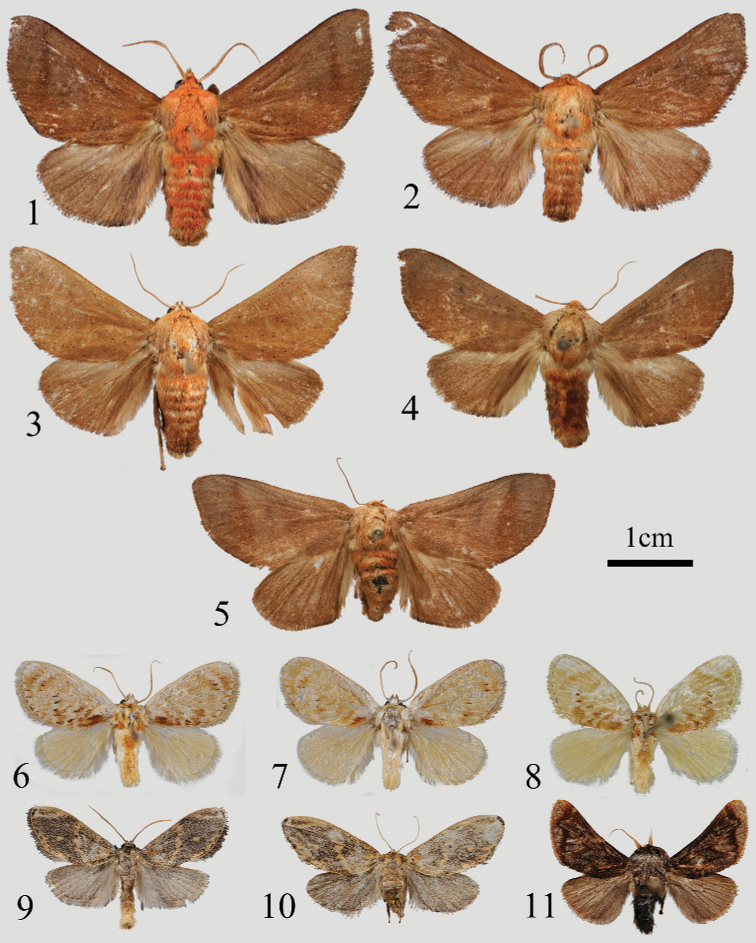
Adults: **1***Iragoidespeiwangi* sp. nov., male, holotype, Xizang, China (NEFU) **2***I.peiwangi* sp. nov., male, paratype, Xizang, China (NEFU) **3***I.elongata* Hering, 1931, male, Chongqing, China (NEFU) **4***I.elongata* Hering, 1931, male, Yunnan, China (NEFU) **5***I.elongata* Hering, 1931, female, Chongqing, China (NEFU) **6***Caelestomorphaserratus* sp. nov., male, holotype, Xizang, China (NEFU) **7***C.serratus* sp. nov., male, paratype, Xizang, China (NEFU) **8***C.endodonta* (Hampson, 1987), male, N.W. India (MWM/ZSM) **9***Euphlyctinaphaeopasta* (Hampson, 1906), male, Xizang, China (NEFU) **10***E.phaeopasta* (Hampson, 1906), female, Xizang, China (NEFU) **11***Mummuaerata* Solovyev & Witt, 2009, male, Xizang, China (NEFU). Scale bar: 1 cm.

**Figures 12–16.**
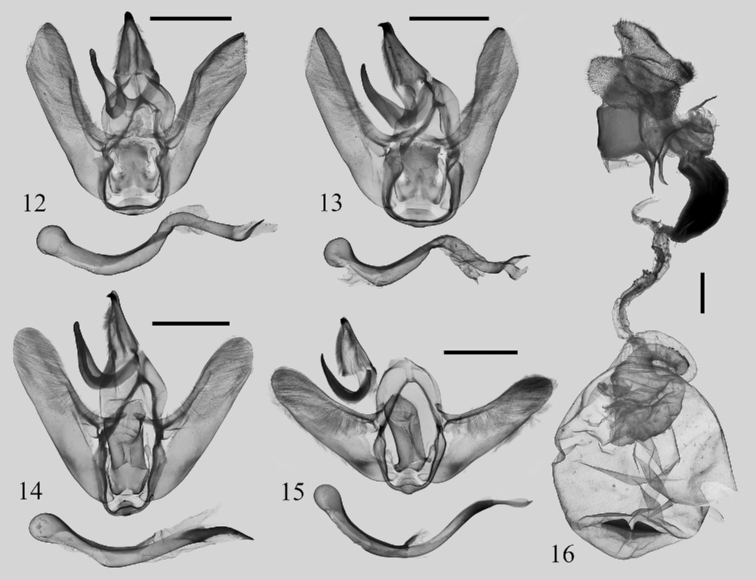
Male genitalia of *Iragoides* spp. **12***I.peiwangi* sp. nov., male, holotype, Xizang, China (NEFU) **13***I.peiwangi* sp. nov., male, paratype, Xizang, China (NEFU) **14***I.elongata* Hering, 1931, male, Chongqing, China, genit. prep. WuJ-536–1 (NEFU) **15***I.elongata* Hering, 1931, male, Yunnan, China, genit. prep. WuJ-086–1 (NEFU) **16***I.elongata* Hering, 1931, female, Chongqing, China, genit. prep. WuJ-708–2 (NEFU). Scale bars: 2 mm.

Figs 1 , 2 , 12 , 13

#### Material examined.

***Holotype*.** ♂, China, Xizang Autonomous Region, Linzhi (= Nyingchi) City, Motuo (= Medog) County, Beibeng Countryside, Dergong Village, 25.V.–4.VI.2021, HL. Han leg., genit. prep. WuJ-523-1 (NEFU). ***Paratypes*.** 5 ♂, China, same data as for holotype, genit. prep. WuJ-524-1 (NEFU).

#### Diagnosis.

The new species *I.peiwangi* sp. nov. (Figs 1, 2) is similar in appearance to *I.elongata* (Figs 3–5), but can be distinguished from the latter by the forewing and male genitalia characters as follows. The forewing of *I.peiwangi* sp. nov. is broad, not elongated; the ground colour is dark reddish brown; the subterminal line is oblique, dark brown and runs from the costal margin near apex to tornus. However, in *I.elongata*, the forewing is elongated; the ground colour is yellowish brown; and the subterminal line is extremely blurred and barely visible.

In the male genitalia, the new species (Figs 12, 13) displays distinct differences with *I.elongata*: the cucullus is sagittate; the upper part of juxta is short, nearly square; the aedeagus is twisted. In *I.elongata* (Figs 14, 15), the cucullus is rounded; the upper part of juxta is long, extending above the upper base of the valvae, and nearly rectangular; the aedeagus is slender and arc-shaped without being twisted.

#### Description.

**Adult** (Figs 1, 2). Forewing length 18–20 mm, wingspan 40–43 mm in male. Head densely covered with reddish orange scales; labial palpus dark brown; antenna shortly pectinate, dark brown, with a distinct silvery basal spot. Thorax mainly reddish orange; tegula pale brown. Forewing broad, not elongated, with a blunt apex; middle of outer margin slightly presented at an obtuse angle; forewing ground colour dark reddish brown, with a dark brown oblique subterminal line that runs from costal margin near apex to tornus which is behind vein Cu1A and almost invisible; discal spot dark, not distinct. Hindwing ground colour brown, dark brown along with the anal margin. Scales on legs dark brown, with distinct slivery spots on distal parts of coxa, femur, tibia and tarsal segments of foreleg. Tibial spurs 0-2-4. Abdomen yellowish brown with an orange-red median band dorsally; ventral side mostly brown with terminus pale brown.

***Male genitalia*** (Figs 12, 13). Uncus triangular with a strongly sclerotized apical spur. Gnathos well developed, hook-shaped, slightly blunt apically. Tegumen broad. Valva narrow, densely covered with hairs; sacculus slightly inflated, without sacculus process; costa with a sclerotized plate-shaped basal process; cucullus sagittate, pointed apically. Juxta flatted with upper part nearly square and basal part with a pair of membranous, finger-shaped lateral processes. Vinculum robust and strongly sclerotized. Saccus not obvious. Aedeagus slender, twisted, slightly inflated at caecum and with a long, strongly sclerotized spur at terminal part.

***Female genitalia*.** Unknown.

#### Bionomics.

The specimens were collected with a light trap at altitudes of ~ 850 m a.s.l in May to June. The collection area is in a subtropical climate zone (Fig. 25).

#### Distribution.

China (Xizang: Motuo).

#### Etymology.

The species is dedicated to Mr. Pei Wang, who works in the People’s Government of Motuo County, and was of great assistance our collecting in Motuo.

### ﻿ Iragoides elongata

Taxon classificationAnimaliaLepidopteraLimacodidae

Hering, 1931

8DE01DF2-9753-57AF-B716-370742BFA02B

Figs 3 , 4 , 5 , 14 , 15 , 16

Iragoides elongata Hering, 1931, in Seitz, Gross-Schmett. Erde 10: 671 (key), 709, fig. 90 f. Type locality: “Hkamkawn, 400 Fuß (Ober-Burma)”. Holotype: ♂ (BMNH).

#### Specimens examined.

1 ♂, China, Prov. Yunnan, Lvchun County, Mt. Huanglian, 27–31.VII.2018, HL. Han and J. Wu legs., genit. prep. WuJ-086-1 (NEFU); 2 ♂, China, Prov. Yunnan, Baoshan City, Mangkuan Town, 30.VII–2.VIII.2014, HL. Han leg., genit. prep. WuJ-698-1 (NEFU);1 ♂, China, Chongqing Municipality, Mt. Simian, 24.VII.–4.VIII.2019, TT. Zhao and SC. Deng legs., genit. prep. WuJ-536-1 (NEFU); 1 ♂, 1 ♀, China, Chongqing Municipality, Mt. Simian, 23.VII.–6.VIII.2018, GX. Wang and WJ. Li legs., genit. prep. WuJ-537-1, WuJ-708-2 (NEFU); 2 ♂, China, Chongqing Municipality, Mt. Simian, 29.VII.–2.VIII.2020, HL. Han and J. Wu legs., genit. prep. WuJ-707-1 (NEFU).

#### Redescription.

**Adult** (Figs 3–5). Forewing length 17–20 mm, wingspan 36–42 mm in male, 21 mm and 42 mm in the single studied female. Head yellowish brown; labial palpus brown; antennae brown with a small silvery basal spot, shortly pectinate in male and filiform in female. Thorax mainly reddish brown, tegula brown. Forewing elongated with acute apex, slightly concaved subapically at outer margin; upper surface without an oblique line and dark discal spot usually indistinct; ground colour yellowish brown with more or less scattered with black scales. Hindwing reddish brown, more yellowish ochreous towards the costal margin. Legs dark brown to yellowish brown; silvery spots are present at distal parts of coxa, femur, tibia and tarsal segments of foreleg. Tibial spurs 0-2-4. Abdomen reddish brown with an orange-red median band dorsally, brown ventrally.

***Male genitalia*** (Figs 14, 15). Uncus triangular, with slender tip and strongly sclerotized apical spur. Gnathos well developed, hook-shaped. Tegumen broad. Valva slender, slightly narrower at terminal part; cucullus rounded. Juxta faintly sclerotized, nearly rectangular, with a pair of membranous, setaceous, finger-shaped lateral processes. Vinculum strongly sclerotized. Saccus not obvious. Aedeagus slender, arc-curved, slightly expanded at caecum then tapering towards terminus; terminal part sclerotized, long spur-shaped. In some individuals, the aedeagus are robust, thicker, and less strongly curved.

***Female genitalia*** (Fig. 16). Papillae anales broad, flattened, densely covered with setae. Anterior apophyses robust, acuate apically; posterior apophyses slender, ca 1.5× the length of anterior apophyses. Genital chamber smooth, sclerotized. Ductus bursae narrow in base; subbasal part swollen, strongly sclerotized; terminal part slender, membranous, slightly twisted. Corpus bursae large, ovoid, covered with tiny granules; signum long and narrow, split into a pair of slightly joined triangular plates at middle, the surface of plate densely covered with fine tooth.

#### Bionomics.

The specimens from Chongqing were collected with a light trap close to a subtropical mixed forest in July to August (Fig. 24).

#### Distribution.

China (Hubei, Guangxi, Sichuan, Chongqing, Yunnan, Xizang), Myanmar, Thailand, northern and central Vietnam.

#### Remarks.

We noticed that the males collected from Chongqing (Figs 3, 14) showed some differences from those collected from Yunnan (Figs 4, 15): the population from Chongqing is distinctly larger in size: the forewing is usually 2 mm and the wingspan 4–5 mm longer than the population from Yunnan; and the aedeagus is thicker, more robust, and less strongly curved. Therefore, we presume that the members from Chongqing possibly represent a new taxon, e.g. a new subspecies. However, given the lack of enough morphological and molecular evidence to prove it, these differences are presently regarded as intraspecific variations.

### ﻿Genus Caelestomorpha

Taxon classificationAnimaliaLepidopteraLimacodidae

Solovyev & Witt, 2009, new genus record for China

77028448-1ACE-58C9-A5DA-2317AB90B44B

Caelestomorpha Solovyev & Witt, 2009, Entomofauna, suppl. 16: 64. Type species (original designation): Caelestomorphaalbiceris Solovyev & Witt, 2009. Type locality Vietnam: Ninh Bình.

#### Remarks.

The genus *Caelestomorpha* was erected by Solovyev and Witt in 2009, with *C.albiceris* Solovyev & Witt, 2009 as its type species. To date, *Caelestomorpha* includes only one congener *C.endodonta* (Hampson, 1897) besides the type species, which was transferred from the genus *Narosa* Walker, 1855 by Solovyev and Witt (2009). Members in this genus are of medium size, the ground colour is pale yellow with a series of dentate, almost parallel, oblique fasciae on the surface of the forewing. Of the male genitalia, the uncus is finger-shaped, blunt apically; the cucullus is densely covered with short hairs; and the vesica contains a field of cornuti (Hampson 1897; Solovyev and Witt 2009). In this paper, the third species *Caelestomorphaserratus* sp. nov. is described below and the genus is newly recorded in China.

### ﻿ Caelestomorpha serratus sp. nov.

Taxon classificationAnimaliaLepidopteraLimacodidae

C4F9E496-474D-5EBD-9D01-1A4DFA2470A3

http://zoobank.org/0949D75A-DE50-4B12-8959-D83DF234B941

**Figures 17–23.**
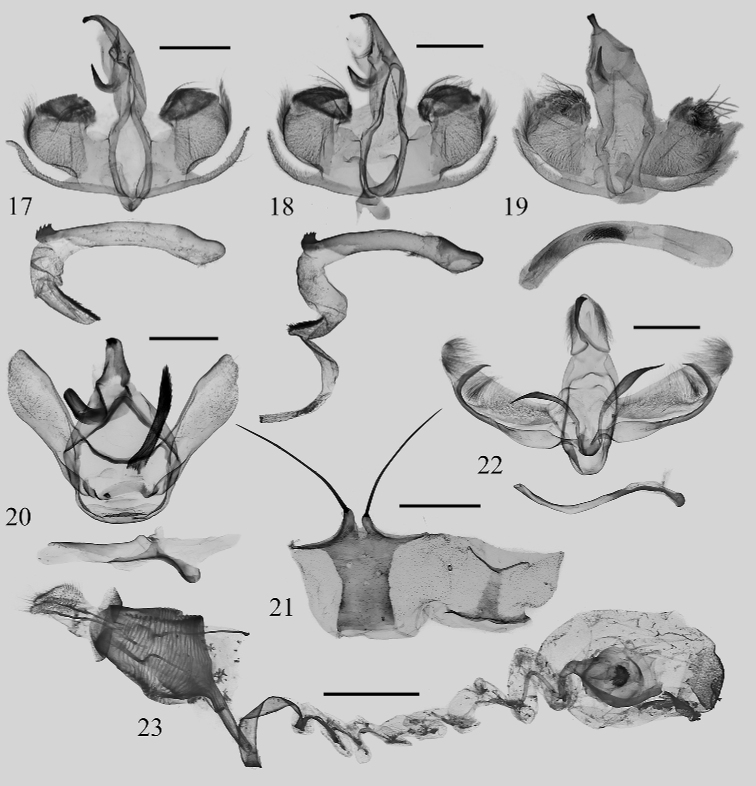
Genitalia and sternite VIII **17***Caelestomorphaserratus* sp. nov., male, holotype (NEFU) **18***C.serratus* sp. nov., male, paratype (NEFU) **19***C.endodonta* (Hampson, 1987), male, N.W. India (MWM/ZSM) **20***Mummuaerata* Solovyev & Witt, 2009, male, Xizang, China, genit. prep. WuJ-551-1 (NEFU) **21–23***Euphlyctinaphaeopasta* (Hampson, 1906): **21** sternite VIII of male, ventral view in left and dorsal view in right (NEFU) **22** male, Xizang, China, genit. prep. WuJ-499-1 (NEFU) **23** female, Xizang, China, genit. prep. WuJ-498-2 (NEFU). Scale bars: 1 mm.

Figs 6 , 7 , 17 , 18

#### Material examined.

***Holotype*.** ♂, China, Xizang Autonomous Region, Linzhi (= Nyingchi) City, Motuo (= Medog) County, Gedang Countryside, 25–30.V.2021, J. Wu and JJ. Fan legs., genit. prep. WuJ-530-1 (NEFU). ***Paratypes*.** 10 ♂, China, same data as for holotype, genit. prep. WuJ-529-1 (NEFU); 14♂, China, Xizang Autonomous Region, Linzhi (= Nyingchi) City, Motuo (= Medog) County, Beibeng Countryside, Dergong Village, 25.V.–4.VI.2021, HL. Han leg., genit. prep. WuJ-490-1 (NEFU).

#### Diagnosis.

The new species is difficult to distinguish from its congener *C.endodonta* (Figs 8, 19) in appearance, but it can be clearly distinguished from the latter by the following characters. In *C.serratus* sp. nov., the yellow colour pattern of the area near forewing apex is more defined (Figs 6, 7); the aedeagus (Figs 17, 18) is slightly curved, with a sawblade-shaped process at the apex; a row of bristle-shaped cornuti located at the terminal area of the aedeagus on the vesica. In *C.endodonta*, however, the yellow band in the apex area of forewing (Fig. 8) is blurry; the curvature of the aedeagus (Fig. 19) is markedly more curved than in the new species and the apex is without a process; *C.endodonta* has a number of cornuti but their position could not be compared with *C.serratus* because the vesica hasn’t been everted.

#### Description.

Adult (Figs 6, 7). Forewing length 12–14 mm, wingspan 25–28 mm in male. Head covered with pale yellow scales; labial palpus slightly up-curved, black to brown at dorsal and lateral sides, pale yellow at ventral side; antenna filiform, brown. Thorax brown mixed with pale yellow; tegula pale yellow. Forewing rounded, ground colour pale yellow, with a whiteish apex; a series of dentate almost paralleled oblique fasciae and yellow bands on forewing surface, and a distinct brown patch along with the inner margin near wing base; terminal line conspicuous, broken, brown; fringe pale brown. Hindwing pale yellow. Legs pale yellow to whitish. Tibial spurs 0-2-4. Abdomen brown to pale brown dorsally; pale yellow ventrally.

***Male genitalia*** (Figs 17, 18). Uncus slender, finger-shaped, blunt and strongly sclerotized apically. Gnathos short, hook-shaped. Valve short and broad, basal part smooth, slightly sclerotized; medial part densely covered with setae, outer margin bearing a large tuft of long hairs; sacculus arc-curved, slightly sclerotized, with a slender, setaceous, nearly membranous sacculus processes; costa extremely shorter than sacculus, strongly recurved; cucullus highly modified, densely covered by short hairs and with a row of very long hairs. Juxta horseshoe-shaped, with a pair of membranous lateral processes. Vinculum thick, slightly sclerotized. Aedeagus tube-shaped, slightly curved; it bears a strongly sclerotized, sawblade-shaped process at terminus, which with 5–6 small tooths; vesica contains a row of strongly sclerotized, bristle-shaped cornuti.

***Female genitalia*.** Unknown.

#### Bionomics.

The specimens were collected with a light trap at altitudes of 1840–2120 m a.s.l. in May to June, close to a subtropical evergreen broad-leaved forest with massive shrubs, ferns and patches of grassland growing in the ground cover layer of the forest (Figs 25–27).

#### Distribution.

China (Xizang: Motuo).

#### Etymology.

The species name is from the Latin “serratus”, which means serrated, notched, alluding to the aedeagus bears a distinct sawblade-shaped process apically.

### ﻿Genus Euphlyctina

Taxon classificationAnimaliaLepidopteraLimacodidae

Hering, 1931

9FEA903D-A606-525A-8DEA-6622661BCBF7

Euphlyctina Hering, 1931, in Seitz, Gross-Schmett. Erde 10: 671 (key), 704. Type species (original designation): Araeogyiaphaeopasta Hampson, 1906. Type locality India: Darjeeling.

#### Remarks.

*Euphlyctina* is a small genus in the family Limacodidae, with only two known species: *E.phaeopasta* (Hampson, 1906) and *E.butvilai* Solovyev & Saldaitis, 2021. The type species *E.phaeopasta* was transferred from the genus *Araeogyia* Hampson, 1893 by Hering (1931), and known only from north India; the second species was discovered in Zhejiang and Yunnan provinces of China (Hampson 1906; Hering 1931; Solovyev and Saldaitis 2021). In 2021, both sexes of *E.phaeopasta* were found in southeastern Xizang; a female of the genus is described below for the first time.

### ﻿ Euphlyctina phaeopasta

Taxon classificationAnimaliaLepidopteraLimacodidae

(Hampson, 1906), new species record for China

B8FE80B5-7CCE-5F53-8CC4-34FE12C162CA

Figs 9 , 10 , 21 , 22 , 23

Araeogyia phaeopasta Hampson, 1906; Proc. Zool. Soc. Lond. 1906: 492, pl. 36, f. 22; Type locality India: Darjeeling. Type: ♂ (ZMHB).
Euphlyctina phaeopasta ; Hering, 1931, in Seitz, Gross-Schmett. Erde 10: 671 (key), 704.

#### Specimens examined.

13 ♂, China, Xizang Autonomous Region, Linzhi (= Nyingchi) City, Motuo (= Medog) County, Beibeng Countryside, Dergong Village, 25.V.–4.VI.2021, HL. Han leg., genit. prep. WuJ-549-1, WuJ-551-1 (NEFU); 7 ♂, 2 ♀, China, Xizang Autonomous Region, Linzhi (= Nyingchi) City, Motuo (= Medog) County, Gedang Countryside, 25.V.–5.VI.2021, J. Wu and JJ. Fan legs., genit. prep. WuJ-498-2, WuJ-499-1 (NEFU); 2 ♂, China, Xizang Autonomous Region, Linzhi (= Nyingchi) City, Pailong Countryside, 7.VI.2021, HL. Han, J. Wu and JJ. Fan legs. (NEFU); 1 ♂, China, Xizang Autonomous Region, Linzhi (= Nyingchi) City, Pailong Countryside, 22.VI.2019, MJ. Qi and JQ. Deng legs., genit. prep. WuJ-436-1 (NEFU).

#### Redescription.

**Adult** (Figs 9, 10). Forewing length 9–11 mm, wingspan 19–23 mm in male; 12 mm and 25 mm in female of two individuals. Head covered by dark brown scales; labial palpus up-curved, dark brown; antenna filiform. Thorax mainly dark brown mixed with black scales; tegula dark brown. Forewing elongated; ground colour ochreous mixed with dark brown; subapical mark nearly triangle, pale brown, embedded with dark brown patch in medial part; apex dark; forewing with an elliptical ochreous spot located between bases of vines M_3_ and Cu1A; an ochreous oblique line runs from middle of costal to inner margin at ca 1/3 distance from wing base; and an ochreous, zig-zag line along with outer margin from vines R_5_ to 1A+2A. Fringe black. Hindwing greyish brown; fringe brown. Fore and mid legs black to dark brown, except tarsus pale brown with long black scales; hindleg pale brown, tarsus same as the former legs. Tibial spurs 0-2-4. Abdomen dark brown to brown, thin and long in male, short and thick in female; sternite VIII with a pair of long and strongly sclerotized piliform processes in male (Fig. 21).

***Male genitalia*** (Fig. 22). Uncus broad with a papilla in apex. Gnathos sickle-shaped, upper half strongly sclerotized, widened apically. Tegumen short and broad. Valva elongated; base of sacculus slightly inflated, with a large sickle-shaped sacculus process located at margin ca 2/3 distance from the base; cucullus slightly narrower, rounded apically, densely covered with hairs. Juxta with a pair of slender, unequal in length, crescent-shaped lateral processes, the right one is a little longer than the left. Saccus broad, tongue-shaped. Aedeagus slender, sinuous.

***Female genitalia*** (Fig. 23). Papillae anales small, flattened, densely covered with short setae. Anterior and posterior apophyses nearly equal in length and both blunt apically. Antevaginal plate sclerotized, with distinctly crumpled surface. Upper part of ductus bursae strongly sclerotized, straight and tube-shaped firstly, then twisted ribbon-shaped, and medial and posterior portions spiral-shaped and membranous. Corpus bursae oval-shaped, with a medial heart-shaped signum.

#### Distribution.

China (Xizang: Motuo), India.

#### Bionomics.

The specimens were collected with a light trap at altitudes of 1840–2200 m a.s.l. in May to June, close to a subtropical evergreen broad-leaved forest with massive shrubs, ferns and patches of grassland growing in the ground cover layer of the forest (Figs 25–27).

#### Remarks.

The genus contains two described species to date since its erected in 1931, but records of the females have been lacking. The female adult and genitalia are described herein for the first time.

### ﻿Genus Mummu

Taxon classificationAnimaliaLepidopteraLimacodidae

Solovyev & Witt, 2009, new genus record for China

76D086B0-C653-5478-A6C5-AF5C9C3B1448

Mummu Solovyev & Witt, 2009, Entomofauna, suppl. 16: 182. Type species (original designation): Mummuaerata Solovyev & Witt, 2009. Type locality N. Vietnam: Lao Cai (Mt. Fan-si-pan W-Seite).

#### Remarks.

The genus *Mummu* is also a small, recently erected genus. The obvious diagnostic features are the forewing with dark brown ground colour, a pale distal region along with the outer margin separated by a narrow darker band with a lighter coloured thin and wavy fascia running from the tornus to the costa near the apex. In the male genitalia, the juxta is strongly modified, the presence of a large, finger-shaped, hairy right process of the juxta, and the valvae without saccular processes are two diagnostic features in this genus. The genus presently includes *M.aerata* Solovyev & Witt, 2009 and *M.cuprea* (Moore, 1879); the latter was transferred from *Pseudonirmides* Holloway, 1986 (Hering 1931; Holloway 1986; Yoshimoto 1994; Solovyev and Witt 2009). We collected four males of *M.aerata* from southeastern Xizang and southwestern Yunnan; it also indicates that the genus occurs in China.

### ﻿ Mummu aerata

Taxon classificationAnimaliaLepidopteraLimacodidae

Solovyev & Witt, 2009

FF948E8F-4ABF-5107-9CD3-A0EBDD8795C4

Figs 11 , 20

Mummu aerata Solovyev & Witt, 2009, Entomofauna, suppl. 16: 183, pl. 15, figs 19–20. Type locality N. Vietnam: Lao Cai (Mt. Fan-si-pan W-Seite). Holotype: ♂ (MWM/ZSM).

#### Specimens examined.

3 ♂, China, Xizang Autonomous Region, Linzhi (= Nyingchi) City, Motuo (= Medog) County, Beibeng Countryside, Dergong Village, 25.V.–4.VI.2021, HL. Han leg., genit. prep. WuJ-549-1, WuJ-551-1 (NEFU); 1 ♂, China, Prov. Yunnan, Pu’er City, Lancang County, Fofang Community, 20.IV.2013, HL. Han leg., genit. prep. WuJ-699-1 (NEFU).

#### Diagnosis.

Based on the descriptions of Solovyev and Witt (2009), *M.aerata* is extremely similar in appearance to its rare Himalayan congener *M.cuprea* (Moore, 1879). The difference between them is that the former has a better-defined border of the distal pale zone in the forewings, whereas the same area in *M.cuprea* is smooth.

#### Bionomics.

The specimens were collected from May to June at altitudes of ~ 850 m a.s.l. The collection area is a subtropical climate zone (Fig. 25).

**Figures 24–27.**
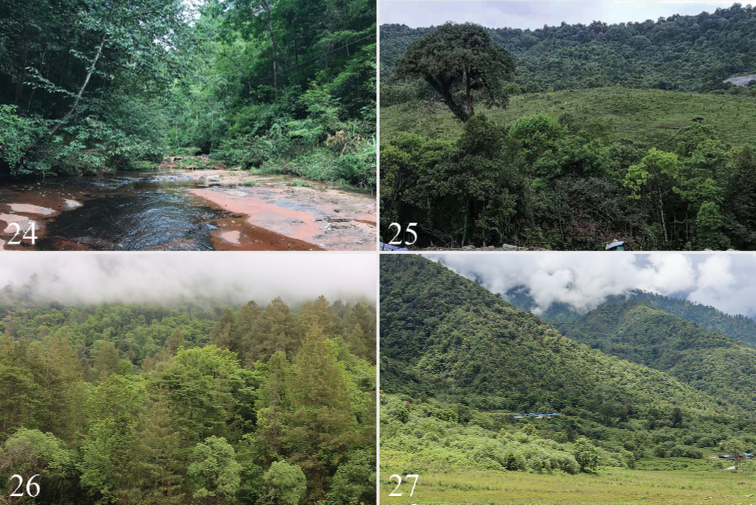
Biotopes: **24** China, Chongqing Municipality, Mt. Simian, biotope of *Iragoideselongata* Hering, 1931, photo by J. Wu **25–27** China, SE Xizang, Linzhi City, Motuo County: **25** Beibeng Countryside, Dergong Village, biotope of *Iragoidespeiwangi* sp. nov., *Caelestomorphaserratus* sp. nov., *Euphlyctinaphaeopasta* (Hampson, 1906) and *Mummuaerata* Solovyev & Witt, 2009, photo by H.L. Han **26, 27** two different collecting sites in Gedang Countryside, biotope of *Caelestomorphaserratus* sp. nov. and *Euphlyctinaphaeopasta* (Hampson, 1906), photo by J. Wu.

#### Distribution.

China (Xizang: Motuo; Yunnan: Lancang), Thailand, Vietnam.

#### Remarks.

The genus is associated with the genera *Pseudonirmides* Holloway, 1986, *Nirmides* Hering, 1931 and *Chibiraga* Matsumura, 1931 both in appearance and the smaller pairwise distances of *COI* (0.097–0.137) (Solovyev 2015); however, the presence of the strongly modified asymmetric juxta with a large, finger-shaped, hairy right process and the absence of the saccular processes of valvae are the diagnostic features.

## ﻿Discussion

Although the most recent species checklist was published by Pan and Wu (2015) with a total of 30 species in 20 genera, we consider the Limacodidae fauna of Xizang to be largely unknown. This is because the known distribution of limacodid moths in Xizang is mainly concentrated in the regions bordering India, Nepal and Bhutan: countries that have a large number of Limacodidae species described or recorded (Hering 1931, 1933; Yoshimoto 1993, 1994; Irungbam et al. 2017). Considering the geographic isolation and climatic conditions of Xizang, there is a high probability that the species beyond the border regions do not overlap with the aforementioned countries.

In addition to the new species and new records mentioned in this study, we still have some materials from Motuo County that cannot be accurately identified at this time. We believe that there is still a large number of species of Limacodidae in Xizang, especially in southern and southeastern regions, that have yet to be discovered. Therefore, more in-depth investigations and studies are needed in the future.

## Supplementary Material

XML Treatment for Iragoides

XML Treatment for Iragoides peiwangi

XML Treatment for Iragoides elongata

XML Treatment for Caelestomorpha

XML Treatment for Caelestomorpha serratus

XML Treatment for Euphlyctina

XML Treatment for Euphlyctina phaeopasta

XML Treatment for Mummu

XML Treatment for Mummu aerata
